# Case report: surgical management of symptomatic pretracheal thyroid gland in a patient with dual ectopic thyroid

**DOI:** 10.1186/s13044-022-00141-5

**Published:** 2022-12-12

**Authors:** Masayuki Saito, Hirona Banno, Yukie Ito, Mirai Ido, Manami Goto, Takahito Ando, Junko Kousaka, Yukako Mouri, Kimihito Fujii, Tsuneo Imai, Shogo Nakano, Toyonori Tsuzuki

**Affiliations:** 1 Division of Breast and Endocrine Surgery, Department of Surgery, Aichi Medical University, 1-1 Yazakokarimata, Aichi 480-1195 Nagakute-City, Japan; 2 Hospital Pathology Department, Aichi Medical University, 1-1 Yazakokarimata, Nagakute-City, Aichi 480-1195 Japan

**Keywords:** Case report, Ectopic thyroid tissue, Dual ectopic thyroid, Surgical management, Cosmetic outcome

## Abstract

**Background:**

Dual ectopic thyroid, a very rare condition, is defined as the simultaneous presence of ectopic thyroid tissue in two abnormal locations. Here, we report the surgical management of a patient with dual ectopic thyroid.

**Case presentation:**

The patient was a 12-year-old boy with right para-midline swelling for 2 months. On physical examination of the upper anterior neck, there was a 4 cm × 3 cm mass that was soft, mobile, smooth, and painless. Blood examination showed euthyroidism. Neck ultrasonography showed a well-circumscribed multilocular cyst. We followed up by observation only because the patient had no local symptoms or malignancy. After 2 years, the mass gradually enlarged, so we performed surgery to improve cosmetic outcomes. Preoperative neck CT revealed both a high-density solid mass at the base of the tongue and a central low-density region surrounded by a high-density area at the pretracheal region below the hyoid. The infrahyoid mass was surgically removed, and the sublingual mass was left intact. Pathological findings showed the growth of multiple-size follicles, leading to a diagnosis of adenomatous goiter. Postoperative ^123−^I scintigraphy showed radioactive iodine uptake in the sublingual lesion, but none in the normal thyroid bed despite the extirpation of thyroid tissue. Postoperative thyroid hormone replacement was started for subclinical hypothyroidism. One year postoperatively, the patient became euthyroid.

**Conclusion:**

Surgical excision was used to manage a symptomatic cervical infrahyoid mass related to dual ectopic thyroid. Postoperatively, thyroid hormone replacement was required both to prevent enlargement of the remaining sublingual thyroid and to maintain adequate thyroid hormone levels.

## Background

Ectopic thyroid tissue (ETT) is a rare embryological anomaly caused by abnormal tissue migration or developmental defects. It is characterized by the presence of thyroid tissue in nonphysiological locations. The prevalence of ETT is estimated to be approximately 1 per 100,000–300,000 people in the general population and is reported to occur in 1 per 4,000–8,000 patients with thyroid dysfunction. ETT is most common in females and is generally observed at a young age [1–4]. The most frequent location of ETT is the base of the tongue [1]. In 70–75% of cases, this lingual thyroid is the only thyroid tissue present [4]. On the other hand, when ETT is located at other sites, an orthotopic thyroid gland is also usually present [4]. Dual ectopic thyroid is the simultaneous presence of ETT in two abnormal locations [1–4]. In most cases, one lesion occurred in the lingual or sublingual area, while the other was in the subhyoid, infrahyoid, or suprahyoid region [4, 5]; furthermore, an orthotopic thyroid gland was extremely rare. Although several authors have reported the surgical treatment of lingual thyroid in ETT, few published reports have described surgery for dual ectopic thyroid. Here, we report a case in which a patient with dual ectopic thyroid was treated surgically.

## Case presentation

**Fig. 1:**
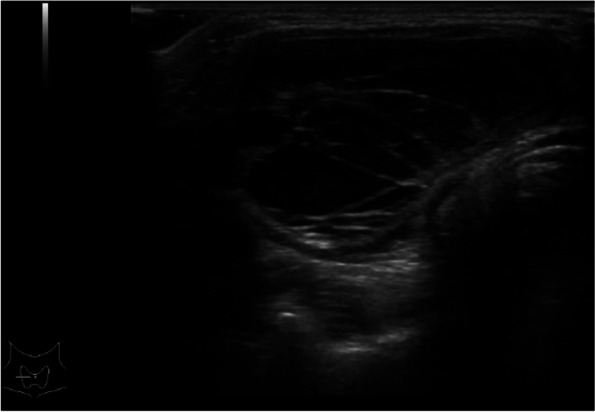
Neck ultrasonography shows a lesion with multiple cysts (4 cm × 3 cm) on the right and atrophy of the left lobe of the thyroid

**Fig. 2:**
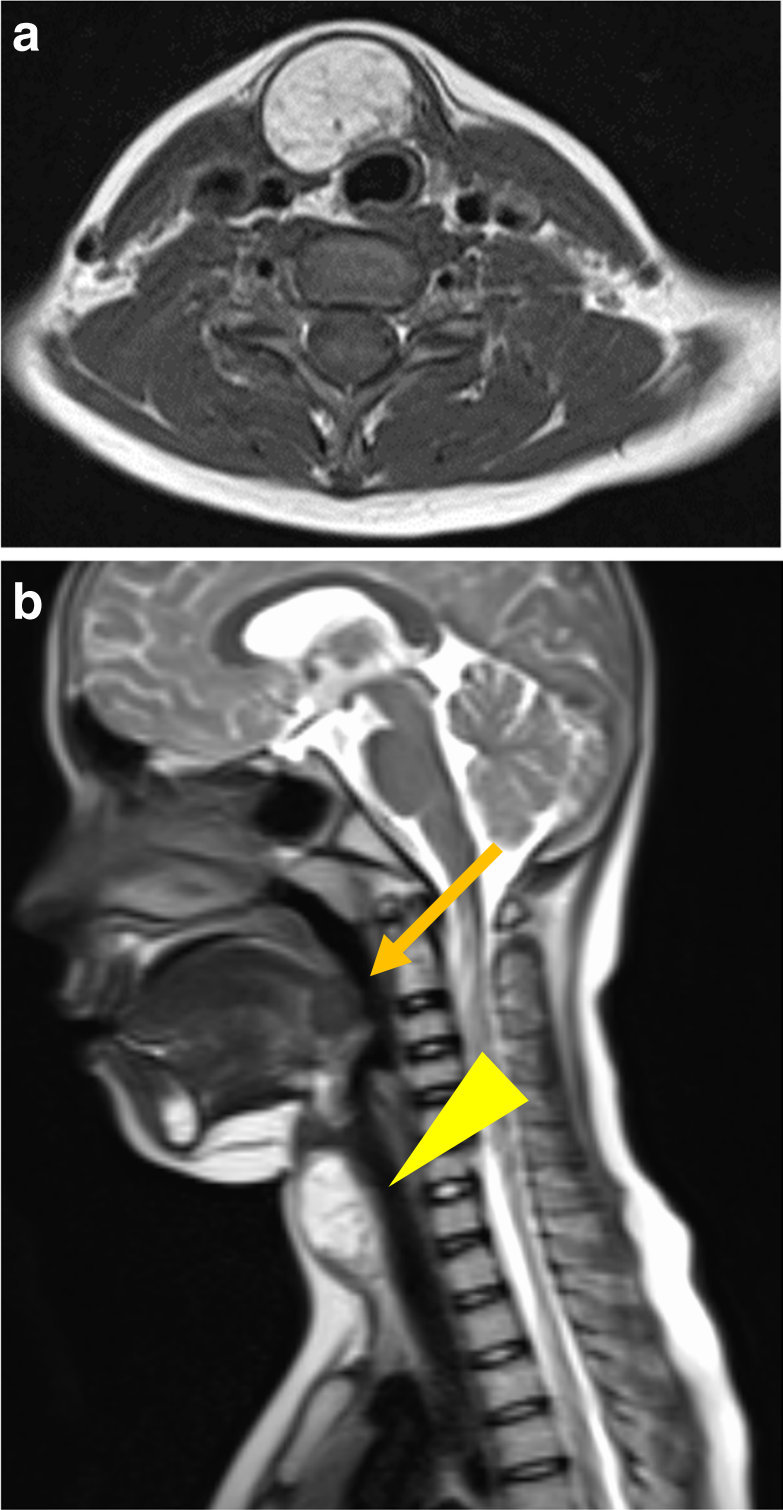
**a**, **b** MRI shows a high-intensity lesion on both T1- and T2-weighted images. The mass showed no suppression on the fat suppression images. The masses were seen in the sublingual lesion (arrow) and pretracheal region below the hyoid (arrow head)

**Fig. 3:**
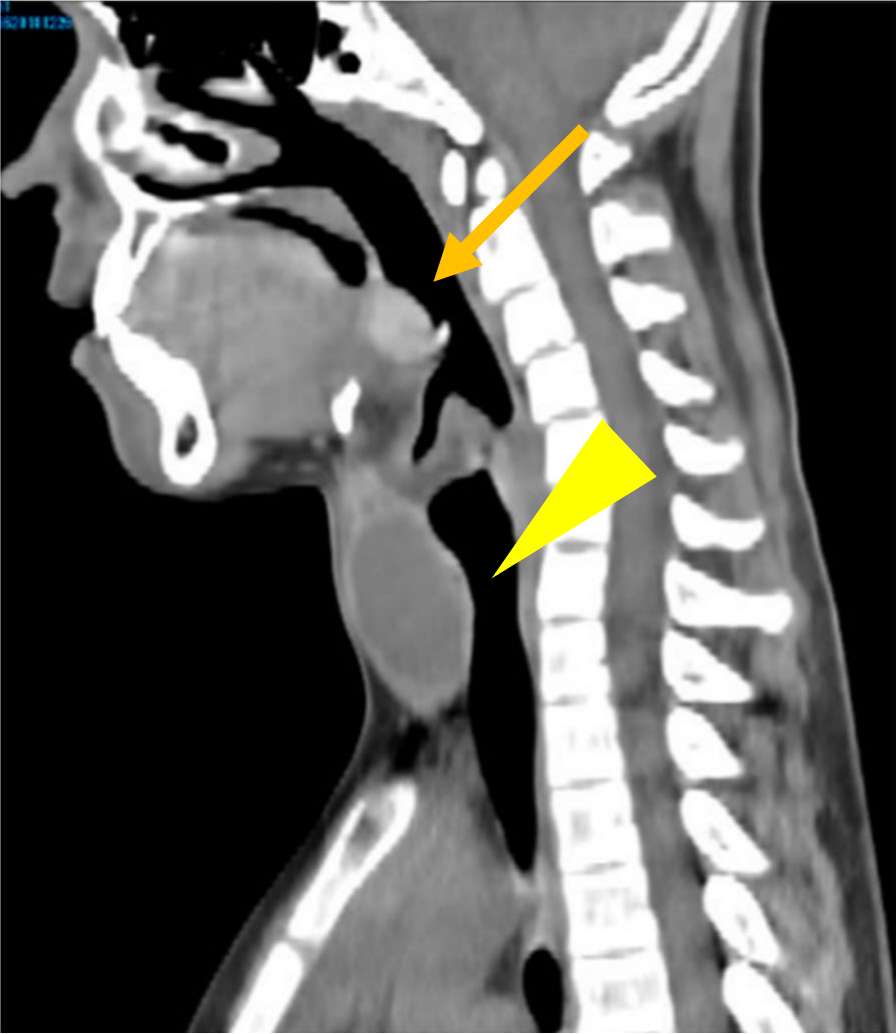
Preoperative CT shows a mass in the sublingual region (arrow) and the development of the pretracheal region below the hyoid (arrowhead)

**Fig.4:**
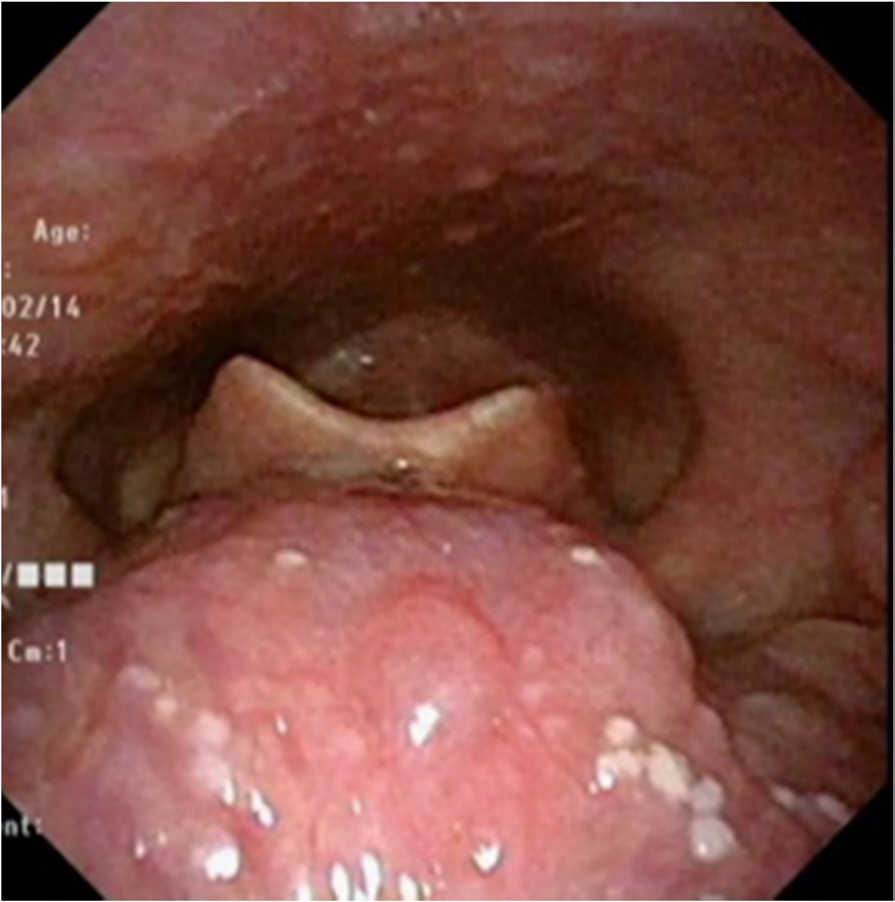
Laryngeal fiberscopy shows the mass (4 cm) in the sublingual lesion

**Fig. 5:**
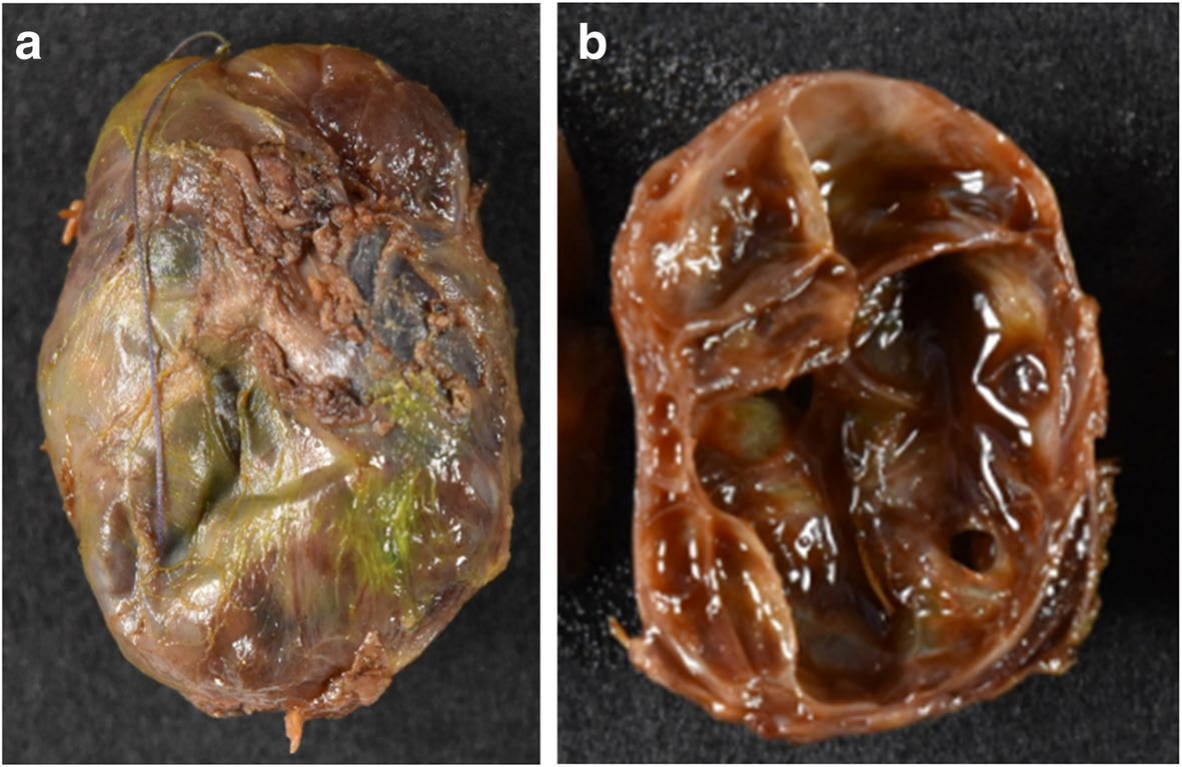
**a**, **b** The pathological specimen demonstrates a lesion with multiple cysts; its size was 6 cm × 5 cm × 4 cm, and its weight was 36 g

**Fig. 6:**
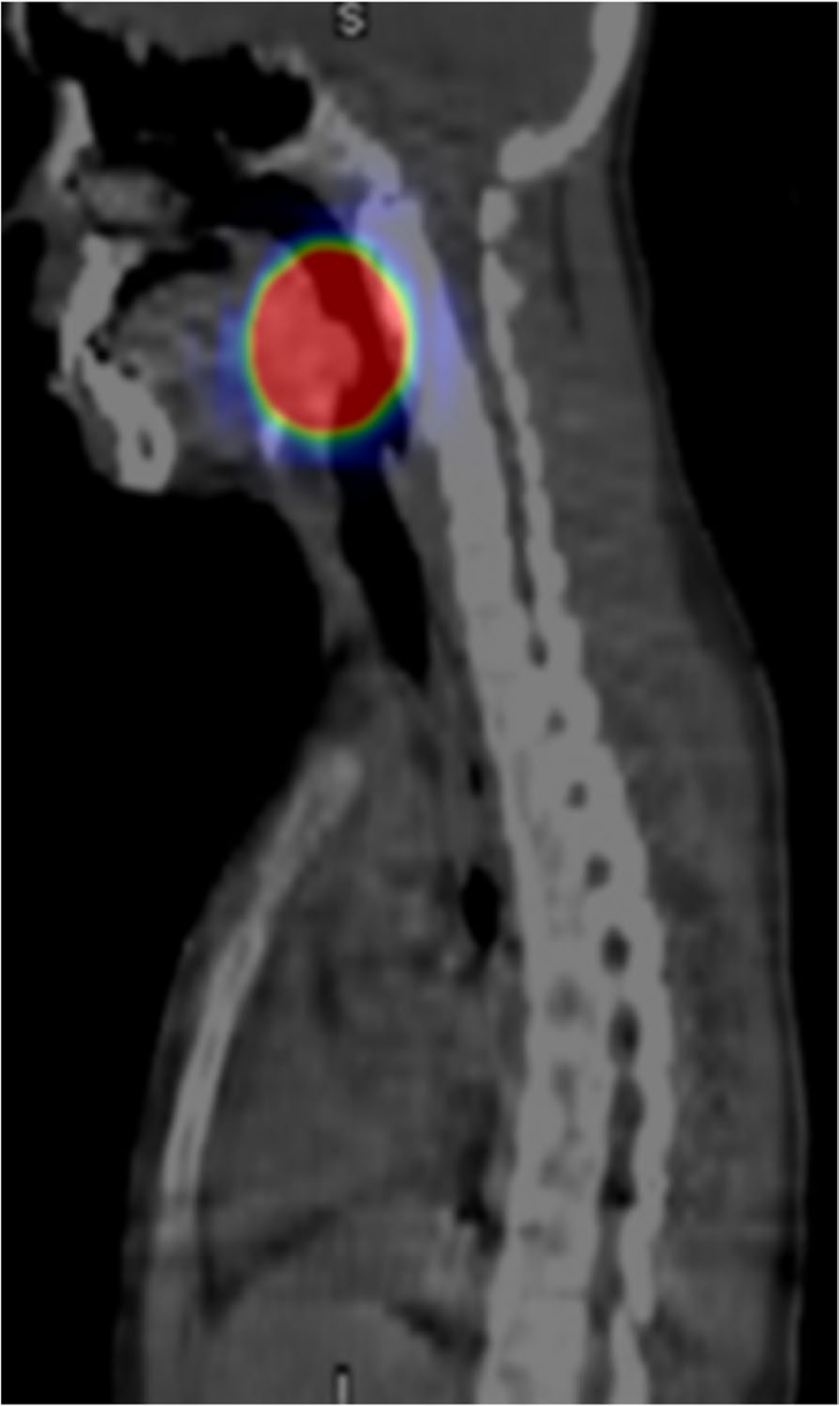
Postoperative ^123−^I scintigraphy shows uptake in the sublingual lesion. This result proved that the sublingual mass was an ectopic thyroid

A 12-year-old boy presented with 2 months of right para-midline neck swelling. Physical examination revealed a soft, mobile, smooth, and painless mass (4 cm × 3 cm in size) in the upper anterior neck. On blood examination, the levels of free T4, thyroid-stimulating hormone, and thyroglobulin (Tg) were 1.03 ng/dl (normal range: 0.70–1.48 ng/dl), 2.399 µU/ml (normal range: 0.350–4.940 µU/ml), and 168.30 ng/dl (normal range: ≤ 33.7 ng/dl), respectively. Anti-Tg antibody was negative. Neck ultrasonography showed a well-circumscribed multilocular cyst (4 cm × 3 cm) in the upper part of the neck, with no orthotopic thyroid gland (Fig. 1). These findings suggested the possibility of undescended thyroid tissue. By neck MRI, both T1- and T2-weighted images showed a round, well-circumscribed lesion of high intensity in the pretracheal region below the hyoid and that of low intensity in the base of the tongue. (Fig. 2a, b). Fine-needle aspiration was performed, and cytological evaluation diagnosed a nonmalignant thyroid cyst. Based on these results, we suspected dual ectopic thyroid with euthyroidism. The patient was followed up every 6 months because he had no local symptoms and was euthyroid. After 2 years, the mass gradually enlarged; thus, we decided to perform surgery to improve the cosmetic outcome. Preoperative neck CT revealed both a high-density solid mass at the base of the tongue and a central low-density region surrounded by a high-density area at the pretracheal region below the hyoid (Fig. 3). Laryngeal fiberscopy showed a mass in the sublingual lesion (Fig. 4). During the operation, the infrahyoid mass, which was well circumscribed with a fibrous capsule, was removed through a low cervical collar incision. The mass was located more cranially than a normal thyroid gland. There was no normal thyroid gland at any location along the trachea. During the surgery, the asymptomatic sublingual mass was left intact. No embryological abnormalities were found in either the parathyroid or recurrent laryngeal nerve. The postoperative course was uneventful, and the patient was discharged on the third day after surgery. The pathological specimen showed a multilocular cystic mass with a size of 6 cm × 5 cm × 4 cm and a weight of 36 g (Fig. 5). Pathological findings showed the growth of multiple-size follicles, which led to a diagnosis of nonmalignant adenomatous goiter. Postoperative ^123−^I scintigraphy showed that radioactive iodine uptake was present in the sublingual lesion, but not in the normal thyroid bed, which was in concordance with the intraoperative findings. (Fig. 6). This result proved that the sublingual mass was ectopic thyroid tissue. Six months postoperatively, blood examination showed subclinical hypothyroidism. The free T4 and TSH levels were 0.84 ng/dl and 15.33 µIU/ml, respectively; thus, replacement therapy with thyroxine 25 µg daily was started to prevent enlargement of the sublingual thyroid. At the time of this writing, 1 year after surgery, the patient’s clinical course was uneventful, and he was euthyroid with thyroxin 50 µg daily.

## Discussion and conclusions

Thyroid tissue develops in the embryo at approximately 4–5 weeks of gestation. It descends from the posterior dorsal midline of the tongue to the region in front of the second to fourth tracheal rings in the neck. Abnormal descent causes ectopic thyroid [4]. The exact mechanism of thyroid morphogenesis is still unclear. Transcription factors appear to play a key role in the organogenesis of the thyroid gland, while TITF 1/NKX2-1 and FOXE1 seem to be involved in thyroid descent and development. Mutations in the corresponding genes may lead to ectopic migration of thyroid tissue [4, 6].

The diagnosis of ETT requires a high index of clinical suspicion. It may or may not be symptomatic, depending on its location. Symptoms include a foreign body sensation, dysphagia, dysphonia, cough, dyspnea, and respiratory obstruction [7]. The differential diagnosis of a cervical mass should include conditions such as epidermal cyst, lymphadenopathy, lymphangioma, lipoma, sebaceous cyst, fibroma, minor salivary gland tumors, midline branchial cysts, and importantly, thyroglossal duct cyst [8]. Regarding thyroid function, approximately half of patients are euthyroid, and the rest are hypothyroid [4, 5]. All diseases capable of affecting scans of the normal thyroid can impact those of ectopic thyroid, including adenoma, hyperplasia, and inflammation, but rarely malignancy [5]. In our case, the mass in the pretracheal region below the hyoid was not malignant.

Regular follow-up is recommended in asymptomatic and euthyroid cases to detect mass enlargement and the development of any complications. However, surgical treatment for ectopic thyroid should only be performed if there are pressure-related symptoms (e.g., neck swelling or airway obstruction) or cosmetic problems related to numerous factors, such as the patient’s age, thyroid function, and complications due to the mass (e.g., ulceration, bleeding, cystic degeneration, or malignancy). When surgical treatment is chosen, postoperative hypothyroidism is an inevitable complication, while potential complications include bleeding, nerve injury, hypoparathyroidism, and surgical site infection. Patients generally require thyroid replacement therapy postoperatively. An extensive search of the literature revealed nine cases of patients who underwent surgery for ectopic thyroid [6–14]. Table 1 shows these cases as well as our own. The median age of the operation was 30.3 years old (range: 15–71) in the nine cases. Ballehaninna UK’s and our patients were the youngest, as far as we have searched, who received the operation. The ratio of males to females was 1 to 8 in the nine cases. The indications for surgery included airway obstruction and an excessively large mass. In seven of nine (78%) patients, thyroid function was normal before surgery but declined thereafter, and replacement therapy was started in seven of the nine (78%) patients postoperatively.

**Table 1:** Summary of nine previously reported patients, including our own, who underwent surgery for ectopic thyroid

Source	Symptom	Age	Sex	Locations	Thyroid function Pre-ope / post-ope	Reason of operation
Hussain D et al.	Neck swelling, dyspnea	42	F	Trachea and nasopharinx	Normal / hypo	Airway obstruction
Saeedi, M et al.	Two huge mass	35	F	Right and left submandibular	Normal / hypo	Huge mass
Ballehaninna UK	Midline neck swelling	15	F	Sublingual and infrahyoid	Hypotyhyroidism / hypothyroidism	Papillarry thyroid carcinoma
Reynaud C et al.	Incidentally detected	28	F	Base of tongue and infrahyoid	Subclinical hypothyroidism / no description	Evaluate the mass
Kwon HJ et al.	Submental mass	37	F	Base of tongue and sublingual	Normal / hypo	Considered to be the thyroglossal duct cyst
Huang TS et al.	Right neck mass	71	F	Lingual and right neck	Normal / hypo	The mass grew gradually
Ulug T et al.	Neck swelling	20	F	Infrahyoid and lingual	Normal / hypo	Cosmetic outcome
Ghanem N et al.	Abnormal screening	24	F	Lingual and porta hepatis	Normal / hypo	Exclude thyroid carcinoma
Hazarika P et al.	Swelling in tongue and chin	32	M	Base of tongue and submandibular	Normal / normal	No description
Our case	Neck swelling	15	M	Base of tongue and right neck	Normal / hypo	Cosmetic outcome

In the case reported here, a symptomatic cervical infrahyoid mass was surgically removed for cosmetic reasons, which was related to dual ectopic thyroid. Postoperatively, thyroid hormone replacement was required both to prevent enlargement of the remaining sublingual thyroid and to maintain proper thyroid hormone levels.

## Data Availability

Not applicable

## Abbreviations

ETT: Ectopic thyroid tissue
MRI: Magnetic resonance imaging
CT: Computed tomography
